# Impact of SARS-CoV-2 on pregnancy and neonatal outcomes: An open prospective study of pregnant women in Brazil

**DOI:** 10.1016/j.clinsp.2022.100073

**Published:** 2022-06-27

**Authors:** Ursula Trovato Gomez, Rossana Pulcineli Vieira Francisco, Fernanda Spadotto Baptista, Maria Augusta B.C. Gibelli, Silvia Maria Ibidi, Werther Brunow de Carvalho, Cristiane de Freitas Paganoti, Ester Cerdeira Sabino, Lea Campos de Oliveira da Silva, Thomas Jaenisch, Philippe Mayaud, Maria de Lourdes Brizot, HC-FMUSP-Obstetric COVID-19 Study Group

**Affiliations:** a Disciplina de Obstetrícia, Departamento de Obstetrícia e Ginecologia, Faculdade de Medicina da Universidade de São Paulo (FMUSP), São Paulo, SP, Brazil; b Hospital Universitário da Universidade de São Paulo (USP), São Paulo, SP, Brazil; c Disciplina de Neonatologia, Departamento de Pediatria, Faculdade de Medicina da Universidade de São Paulo (FMUSP), São Paulo, SP, Brazil; d Departamento de Moléstias Infecciosas e Parasitárias, Instituto de Medicina Tropical, Faculdade de Medicina da Universidade de São Paulo (FMUSP), São Paulo, SP, Brazil; e Laboratório de Medicina Laboratorial (LIM-03), Hospital das Clínicas da Faculdade de Medicina da Universidade de São Paulo (FMUSP), São Paulo, SP, Brazil; f Center for Global Health, Colorado School of Public Health, Aurora, CO, USA; g Heidelberg Institute for Global Health (HIGH), Heidelberg University Hospital, Germany; h Faculty of Infectious & Tropical Diseases, London School of Hygiene & Tropical Medicine, UK

**Keywords:** COVID-19, SARS-CoV-2 Infection, Outcome, Obstetric, Neonatal

## Abstract

**Objectives:**

To determine the incidence and risk of adverse obstetric and neonatal outcomes according to SARS-CoV-2 infection severity in pregnant women.

**Method:**

Open prospective study of pregnant women tested for SARS-CoV-2 by serological and molecular assays during pregnancy or delivery in two hospitals in Sao Paulo, Brazil from April 12, 2020, to February 28, 2021. Five groups were considered for analysis: C0, negative COVID-19 results and no COVID-19 symptoms; C1, positive COVID-19 results, and no symptoms; C2, positive COVID-19 results with mild symptoms; C3, positive COVID-19 results with moderate symptoms; and C4, positive COVID-19 results with severe symptoms. The association between obstetric and neonatal outcomes and COVID-19 severity was determined using multivariate analysis.

**Results:**

734 eligible pregnant women were enrolled as follows: C0 (*n* = 357), C1 (*n* = 127), C2 (*n* = 174), C3 (*n* = 37), and C4 (*n* = 39). The following pregnancy and neonatal outcomes were associated with severe COVID-19: oligohydramnios (adjusted Odds Ratio [aOR] = 6.18; 95% CI 1.87‒20.39), fetal distress (aOR = 4.01; 95% Confidence Interval [CI] 1.84‒8.75), preterm birth (aOR = 5.51; 95% CI 1.47‒20.61), longer hospital stay (aOR = 1.66; 95% CI 1.36‒2.02), and admission to the neonatal intensive care unit (aOR = 19.36; 95% CI, 5.86‒63.99). All maternal (*n* = 6, 15.4%, *p* < 0.001) and neonatal (*n* = 5, 12.5%, *p* < 0.001) deaths and most fetal deaths (*n* = 4, 9.8%, *p* < 0.001) occurred in C4 group. Moderate COVID-19 was associated with oligohydramnios (aOR = 6.23; 95% CI 1.93‒20.13) and preterm birth (aOR = 3.60; 95% CI 1.45‒9.27). Mild COVID-19 was associated with oligohydramnios (aOR = 3.77; 95% CI 1.56‒9.07).

**Conclusion:**

Adverse pregnancy and neonatal outcomes were associated with maternal symptomatic COVID-19 status, and risk increased with disease severity.

## Highlights

• COVID-19 increases the rates of adverse pregnancy and neonatal outcomes.
• Serious cases are associated with oligohydramnios, fetal distress, prematurity, neonatal ICU admission, maternal and neonatal deaths.
• The maternal clinical status dictates obstetric and neonatal outcomes.

## Introduction

Coronavirus 2019 (COVID-19) is a new respiratory and multiorgan illness caused by infection with the novel Severe Acute Respiratory Syndrome Coronavirus type-2 (SARS-CoV-2), which emerged in December 2019 in Wuhan, China. Since it was declared a global pandemic in March 2020, the virus has affected an increasing number of people worldwide, accounting for over four million deaths thus far.^1^ Although pregnancy is a risk factor for more severe infection, ^2^ the disease does not seem to be more aggressive towards this group when compared with non-pregnant women with similar demographic characteristics.^3^^,^^4^ When infection occurs, the vast majority of mothers remain asymptomatic or present mild symptoms, and recovery is usually achieved without the need for delivery.^5^

However, more serious cases of infection, albeit rare, should not be ignored.^6^ It is believed that severe infections are more prevalent in high-risk pregnancies, compared with low-risk pregnancies, and carry the potential for more severe results.^7^ In addition, the severity of the clinical condition is directly associated with obstetric and neonatal outcomes.^8^^,^^9^ Some studies have shown that seriously affected pregnant women have a higher incidence of preterm births,^6^^,^^9^, ^10^, ^11^ cesarean delivery, ^12^^,^^13^ lower Apgar scores, greater admission to the Intensive Care Unit (ICU), ^10^ and neonatal death.^14^ However, recent systematic reviews have not confirmed these or similar associations.^15^^,^^16^

Because of this conflicting evidence, studies that aim to correlate not only the presence of infection but also the severity of the clinical status with adverse obstetric and perinatal outcomes are necessary for better care and counseling of affected women and their families. Therefore, the authors sought to determine the factors associated with adverse pregnancy and neonatal outcomes in this open prospective study.

## Materials and methods

### Population and settings

The data used in the present investigation are part of a cross-sectional nested study at Hospital das Clínicas (HC) and Hospital Universitário (HU), and Sao Paulo University. A detailed description of this study has been published elsewhere.^17^ In brief, at the beginning of the COVID-19 pandemic in São Paulo (March 2020), the two hospitals belonging to the same institution were deliberately separated into two sites: HC was the designated specialist center for the management and admission of COVID-19 patients, while HU received patients not suspected of having COVID-19. In routine practice, HC is a tertiary referral hospital where high-risk pregnancies are referred and followed up during antenatal care and delivery, whereas HU focuses on the delivery of low-risk pregnant women and the management of simpler obstetric procedures, such as dilation and curettage/manual intrauterine aspiration. During the pandemic, however, high-risk pregnancies with no COVID-19, normally seen in HC, were being followed at HU, while apparent low-risk pregnancies with COVID-19 were seen in HC.

### Data collection

Pregnant women admitted to HC due to COVID-19 or those who delivered in both hospitals between April 12, 2020 and February 28, 2021, who fulfilled the following criteria, were selected for the analysis: having been investigated for SARS-CoV-2 (by serology or molecular test) during pregnancy or at delivery, absence of fetal malformation, and known pregnancy and neonatal outcomes.

Women admitted to the Labor & Delivery ward at HU were invited to participate in the study, and those who agreed to participate were given a questionnaire regarding demographics and clinical and obstetric history. The questionnaire also included questions about COVID-19 symptoms and/or flu-like symptoms during pregnancy, COVID-19 diagnosis during pregnancy, need for hospital admission, need for supplemental oxygen, and admission to the Intensive Care Unit (ICU). Women's antenatal records were checked for specific antenatal care information regarding adverse pregnancy outcomes, as described below. Maternal blood was drawn for serological SARS-CoV-2 status investigation.

Women admitted to the HC group were investigated for COVID-19 at hospital admission and answered the same questionnaire as those at HU. All information obtained was entered into REDCap electronic data capture tools, ^18^ hosted at HC. Data regarding pregnancy and neonatal outcomes were obtained from the obstetrics department, neonatal unit, and hospital records and were inserted into the REDCap.

### Laboratory methods

Demonstration of SARS-CoV-2 infection was performed using serological and molecular assays, as previously described.^17^

Serum samples from all participants were tested for SARS-CoV-2 antibodies using Elecsys anti-SARS-CoV-2 E2G300 (Roche Diagnostics, Mannheim, Germany), a chemiluminescence assay that allows the qualitative detection of specific SARS-CoV-2 antibodies. The assay uses a recombinant protein from the Nucleocapsid (N) antigen of the virus with a double-antigen sandwich methodology, which favors the detection of high-affinity antibodies against SARS-CoV-2.

Respiratory tract (nasopharynx and/or trachea) samples from women presenting with suspected COVID-19 symptoms were tested by RT‐PCR (RealStar SARS‐CoV‐2 RT‐PCR kit 1.0 RUO, Altona Diagnostics) using a LightCycler 96 Instrument (Roche).

### Statistical analyses

For the current analysis, the study population was divided into five groups with increasing COVID-19 severity: C0, women with negative serology at delivery and no symptoms during the pregnancy; C1, women with positive serology at delivery or COVID-19 diagnosis during pregnancy with no symptoms; C2, women with positive serology at delivery or COVID-19 diagnosis during pregnancy with mild symptoms; C3, women with COVID-19 during pregnancy with hospital admission and moderate symptoms; C4, women who acquired COVID-19 during pregnancy and required hospital admission for severe COVID-19. Moderate symptoms were defined as a hospital admission with the need for supplemental oxygen delivered by a simple catheter (SpO_2_ <94% and/or respiratory rate > 24 breaths per minute), ^19^ and severe COVID-19 was defined as a hospital admission with the need for admission in the ICU (i.e., supplemental oxygen delivered in forms other than simple catheter [e.g., ventilation] and/or organ involvement).

Adverse pregnancy outcomes were defined as any of the following: preeclampsia, gestational diabetes, miscarriage, fetal distress (defined as either reverse end-diastolic flow of umbilical arteries and/or Pulsatility Index for Veins (PIV) greater than 1.5 in ductus venosus in dopplervelocimetric evaluation and/or final score of biophysical profile < 6 and pathological recurrent decelerations and/or sustained fetal bradycardia in cardiotocography), Preterm Premature Rupture of Membranes (PPROM), oligohydramnios (defined as amniotic fluid index ≤ 5 cm or single deepest vertical pocket ≤2 cm), fetal death, preterm delivery stratified according to gestational age (< 37, < 34, < 32, and < 28 weeks), cesarean section, intrapartum or postpartum atony (defined as absence of uterine contraction with need of uterine massage and additional use of uterotonic drugs [oxytocin] at higher doses than the 10 UI routinely used, or any further intervention for uterine contraction), length of hospital stay after delivery, and maternal death.

Adverse neonatal outcomes were defined as any of the following outcomes: low birth weight classified below the 10th percentile and birth weight below the 3rd percentile for gestational age at delivery, Apgar score of < 7 at 5 min, need for supplemental oxygen, mechanical ventilation, admission to the Neonatal Intensive Care Unit (NICU), length of hospital stay, and neonatal death.

Categorical variables are presented as absolute frequencies and percentages. Continuous variables are presented as medians with interquartile ranges. The Chi-Square test, Fisher's Exact test and linear-by-linear association were used to check the association of categorical variables. Mann-Whitney and Kruskal-Wallis tests were used to assess the differences between groups according to quantitative variables. Multivariate multinomial logistic regression analysis was applied to the variables with significance in the univariate analysis to estimate the Odds Ratio (OR) and 95% Confidence Interval (CI) of adverse outcomes for each group compared with the reference group. The OR in multivariate analysis was adjusted for confounding factors. Differences were considered significant when the p-value was less than 0.05.

Data were analyzed using the Statistical Package for the Social Sciences (SPSS version 20, IBM, Armonk, NY, USA).

## Ethics

The study protocol was approved by the ethics committees of both hospitals (CAAE: 30270820.3.0000.0068) and registered at ClinicalTrial.gov (NCT04647994). All participants provided informed consent prior to participating in the study.

## Results

**Fig. 1:**
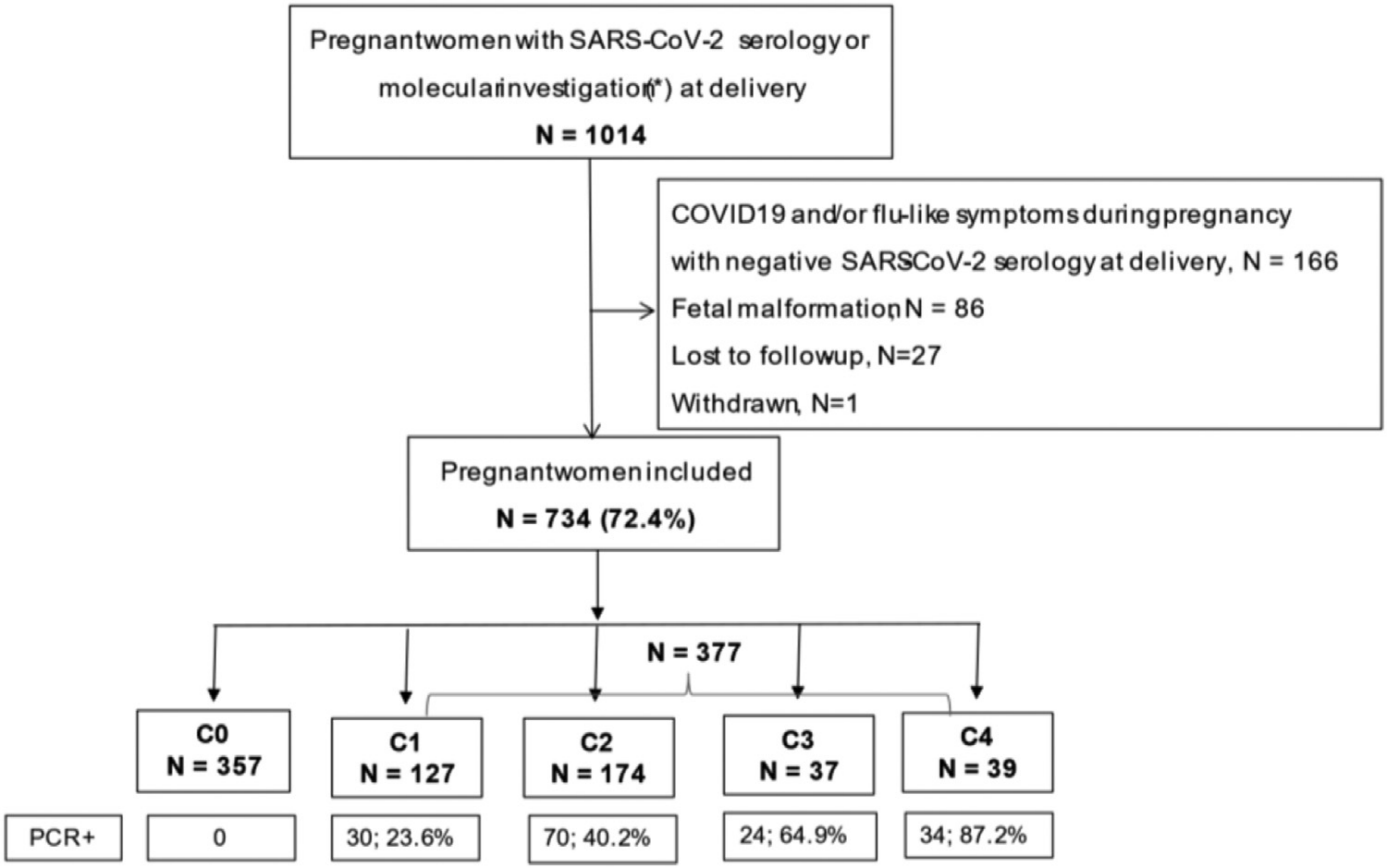
CONSORT flowchart for study inclusions. Nasopharynx Swab investigation (RT-PCR).

During the 11-month study period, 1014 pregnant women had serology or swab investigations for SARS-CoV-2 at delivery, and 734 fulfilled the inclusion criteria for this analysis, with the following distribution according to the COVID-19 severity groups: C0 = 357, C1 = 127, C2 = 174, C3 = 37, and C4 = 39 (Fig. 1). Slightly above 50% (*n* = 377) of the study population tested positive for SARS-CoV-2 infection. The proportion of women who tested positive with molecular tests ranged from 23.6% to 87.2%, with increasing positivity by severity group.

Table 1 shows the characteristics of the infected and uninfected groups. Diabetes mellitus was the only maternal characteristic found to be more frequent among women with SARS-CoV-2 overall (*p* = 0.009). However, the number of maternal characteristics differed by severity (Table 2). Maternal age was higher with increasing disease severity (*p* = 0.044), and inversely, the proportion of nulliparous women was greater among those with less disease severity (*p* < 0.001). Further, Body Mass Index (BMI) was greater (*p* = 0.012), and a history of chronic hypertension was more frequent among women with moderate and severe COVID-19 (*p* < 0.001). Similarly, diabetes mellitus was more frequent among women with COVID-19 symptoms (from C2 to C4 [*p* = 0.013]).

**Table 1:** Population characteristics according to COVID-19 infection (positive or negative) status during pregnancy.

Characteristics	COVID-19 Positive, *n* = 377	COVID-19 Negative, *n* = 357	*p*-value
	*n* (%) or median (IQR)		
Maternal age (years)	28 (24‒33)	29 (24‒35)	0.3^a^
BMI (kg/m^2^)	31 (28‒35), *n* = 370	30.48 (26.9‒35.02), *n* = 351	0.09^a^
Years of schooling	12 (10‒12), *n* = 291	12 (10‒12), *n* = 356	0.21^a^
Smoking during pregnancy	32/374 (9.3)	33/356 (9.3)	0.73^b^
**Obstetric history**			
Nulliparous	140 (37.0)	145/356 (40.7)	0.30^b^
Multiple pregnancy	15 (4.0)	7 (2.0)	0.11^b^
**Comorbidities**			
Diabetes mellitus	18 (4.8)	5 (1.4)	0.009^b^
Chronic hypertension	36 (9.5)	21 (5.9)	0.06^b^
Cardiac disease	16 (4.2)	9 (2.5)	0.20^b^
Lung disease	40 (10.6)	24 (6.7)	0.06^b^
Rheumatic disease	7 (1.9)	5 (1.4)	0.63^b^
Thromboembolic disease	3 (0.8)	5 (1.4)	0.49^c^
**Gestational age at COVID-19 Symptoms (weeks)**	30 (22‒34), *n* = 187	NA	NA

IQR, Interquartile Range; BMI, Body Mass Index; NA, Not Applicable.

a Mann-Whitney Non-Parametric Test.

b Chi-Square Test.

c Fisher´s Exact Test.

**Table 2:** Population characteristics according to COVID-19 severity during pregnancy.

Characteristics	C0, *n* = 357	C1, *n* = 127	C2, *n* = 174	C3, *n* = 37	C4, *n* = 39	*p*-value
	*n* (%) or median (IQR)					
**Demographics**						
Maternal age (years)	29 (24‒35)	28 (23‒32)	27 (23.75‒33.25)	31 (26.5‒34)	30 (26‒36)	0.044^a^
BMI (kg/m^2^)	30 (27‒35), *n* = 351	32 (28‒35), *n* = 122	30 (27‒34)	33 (29‒38), *n* = 36	33 (29.75‒35.75), *n* = 38	0.012^a^
Years of schooling	12 (10‒12), *n* = 356	12 (10‒12), *n* = 125	12 (10‒12), *n* = 128	12 (10‒12), *n* = 20	12 (10‒12), *n* = 18	0.26^a^
Smoking during pregnancy	33/356 (9.3)	14 (11.0)	14/171 (8.2)	2 (5.4)	2 (5.1)	0.81^c^
**Obstetrical history**						
Nulliparous	145/356 (40.7)	52 (40.9)	76 (43.6)	6 (16.2)	6 (15.4)	< 0.001^b^
Multiple pregnancy	7 (2.0)	6 (4.7)	6 (3.4)	1 (2.7)	2 (5.1)	0.32^c^
**Comorbidities**						
Diabetes mellitus	5 (1.4)	3 (2.4)	10 (5.7)	3 (8.1)	2 (5.1)	0.013^c^
Chronic Hypertension	21 (5.9)	8 (6.3)	10 (5.7)	10 (27.0)	8 (20.5)	< 0.001^c^
Cardiac disease	9 (2.5)	4 (3.1)	7 (4.0)	2 (5.4)	3 (7.7)	0.33^c^
Lung disease	24 (6.7)	12 (9.4)	18 (10.3)	7 (18.9)	3 (7.7)	0.13^c^
Rheumatic disease	5 (1,4)	0 (0)	7 (4.0)	0 (0)	0 (0)	0.10^c^
Thromboembolic disease	5 (1.4)	1 (0.8)	1 (0.6)	1 (2.7)	0 (0)	0.66^c^
**Gestational age at COVID-19 symptoms (weeks)**	NA	NA	30 (22‒36), *n* = 118	30 (27‒34), *n* = 31	30.5 (23‒33), *n* = 36	0.95^a^

IQR, Interquartile Range; BMI, Body Mass Index; NA, Not Applicable; C0, Women with negative serology at delivery and no symptoms during pregnancy (reference group); C1, Women with positive serology at delivery or COVID-19 diagnosis during pregnancy with no symptoms; C2, Women with positive serology at delivery or COVID-19 diagnosis during pregnancy with mild symptoms; C3, Women diagnosed with COVID-19 during pregnancy with hospital admission and moderate symptoms; C4, Women diagnosed with COVID-19 during pregnancy with hospital admission and severe COVID-19.

a Kruskal-Wallis Non-Parametric Test.

b Chi-square Test.

c Fisher´s Exact Test.

Multivariate multinomial regression analysis of maternal factors associated with COVID-19 severity (Table 3) showed significant associations for chronic hypertension with moderate COVID-19 (adjusted odds ratio [aOR] = 5.21; 95% CI 2.09‒13.0) and history of diabetes mellitus with mild and moderate COVID-19 (aOR = 4.27; 95% CI 1.43‒12.71; and aOR = 6.23; 95% CI 1.37‒28.34, respectively).

**Table 3:** Association of maternal factors with COVID-19 severity.

	C1, *n* = 127	C2, *n* = 174	C3, *n* = 37	C4, *n* = 39
	OR (95%CI)	OR (95%CI)	OR (95%CI)	OR (95%CI)
**BMI, (kg/m^2^)**^a^				
Crude OR	1.03 (0.99‒1.06)	0.99 (0.96‒1.02)	1.06 (1.01‒1.12)	1.07 (1.02‒1.13)
Adjusted	1.03 (0.99‒1.06)	0.99 (0.96‒1.02)	1.03 (0.97‒1.09)	1.05 (1.00‒1.11)
**Nulliparous**				
Crude OR	1.01 (0.67‒1.52)	1.12 (0.78‒1.61)	0.28 (0.12‒0.69)	0.27 (0.11‒0.65)
Adjusted	0.89 (0.57‒1.38)	1.05 (0.71‒1.56)	0.29 (0.11‒0.76)	0.28 (0.11‒0.72)
**Diabetes mellitus**^a^				
Crude OR	1.70 (0.40‒7.23)	4.27 (1.44‒12.68)	6.21 (1.42‒27.13)	3.81 (0.71‒20.31)
Adjusted	1.69 (0.40‒7.18)	4.27 (1.43‒12.71)	6.23 (1.37‒28.34)	3.58 (0.66‒19.57)
**Chronic Hypertension**^a^				
Crude OR	1.08 (0.46‒2.49)	0.97 (0.45‒2.11)	5.93 (2.54‒13.85)	4.13 (1.69‒10.9)
Adjusted	0.96 (0.40‒2.28)	1.02 (0.46‒2.26)	5.21 (2.09‒13.0)	2.65 (0.99‒7.13)
**Gestational age at COVID‒19 symptoms (weeks)**	NA	Reference	1.03 (0.98‒1.08)	1.01 (0.97‒1.06)

BMI, Body Mass Index; NA, Not Applicable; OR, Odds Ratio; C0, Women with negative serology at delivery and no symptoms during pregnancy (reference group); C1, Women with positive serology at delivery or COVID-19 diagnosis during pregnancy with no symptoms; C2, Women with positive serology at delivery or COVID-19 diagnosis during pregnancy with mild symptoms; C3, Women diagnosed with COVID-19 during pregnancy with hospital admission and moderate symptoms; C4, Women diagnosed with COVID-19 during pregnancy with hospital admission and severe COVID-19.

a Adjusted for confounding factors: diabetes mellitus, chronic hypertension, and BMI.

The occurrence of obstetrical and neonatal outcomes by COVID-19 category and associations of each category compared with those of uninfected women (C0) in uni- and multivariate multinomial analyses are presented in Table 4 and Table 5, respectively.

**Table 4:** Pregnancy outcomes according to maternal COVID-19 infection status.

Outcomes	C0, *n* = 357	C1, *n* = 127			C2, *n* = 174		C3, *n* = 37		C4, *n* = 39		*p*‒value
	Reference	*n* (%), M (IQR)		Adjusted OR (95% CI)	*n* (%), M (IQR)	Adjusted OR (95% CI)	*n* (%), M (IQR)	Adjusted OR (95% CI)	*N* (%), M (IQR)	Adjusted OR (95%CI)	
Preeclampsia	56/343 (16.3)	14/125 (11.2)		NA	24 (13.8)	NA	5/36 (13.9)	NA	5 (12.8)	NA	0.37⁎⁎
Gestational Diabetes^a^	107/343 (31.2)	31/125 (24.8)		0.75 (0.46‒1.23)	35/173 (20.2)	0.59 (0.37‒0.93)	5/36 (13.9)	0.40 (0.15‒1.08)	3 (7.7)	0.75 (0.46‒1.23)	<0.001⁎⁎
Miscarriage	25 (7.0)	7 (5.5)		NA	0	NA	0	NA	0	NA	<0.001⁎⁎⁎
Fetal distress#^,^^b^	34/343 (9.9)	14 (11.0)		1.15 (0.59‒2.21)	23/179 (12.8)	1.31 (0.74‒2.31)	4/38 (10.5)	1.04 (0.35‒3.12)	12 (30.7)	4.01 (1.84‒8.75)	0.012⁎⁎⁎
PPROM	12/336 (3.6)	9/120 (7.5)		NA	16/173 (9.2)	NA	1/36 (2.8)	NA	3/38 (7.8)	NA	0.063⁎⁎⁎
Oligohydramnios#^,^^b^	8/365 (2.7)	4/133 (3.3)		1.37 (0.40‒4.63)	15/181 (8.3)	3.77 (1.56‒9.07)	5/38 (13.2)	6.23 (1.93‒20.13)	5/41 (12.2)	6.18 (1.87‒20.39)	<0.001⁎⁎⁎
Fetal death#^,^^b^	2/340 (0.6)	1/126 (0.8)		NA	2/180 (1.1)	0.99 (0.1‒11.01)	0/38	NA	4/41 (9.8)	4.42 (0.36‒54.80)	0.006⁎⁎⁎
GAD, weeks^a^	39 (38‒40)	39 (38‒40)		0.97 (0.86‒1.09)	39 (37‒40)	0.91 (0.82‒0.99)	39 (36.5‒40)	0.84 (0.73‒0.97)	34 (31‒39)	0.75 (0.64‒0.90)	<0.001*
PD < 37 weeks^a^	23/331 (6.9)	10/118 (8.5)		1.18 (0.54‒2.60)	30/172 (17.4)	1.80 (0.95‒3.44)	9 (24.3)	3.60 (1.45‒9.27)	23/35 (65.7)	5.51 (1.47‒20.61)	<0.001⁎⁎
PD < 34 weeks^a^	7/331 (2.1)	3/118 (2.5)		1.04 (0.26‒4.19)	11/172 (6.4)	1.54 (0.51‒4.63)	3 (8.1)	2.38 (0.54‒10.57)	20/39 (51.3)	9.06 (1.95‒42.21)	<0.001⁎⁎⁎
PD < 32 weeks^a^	2/331 (0.6)	3/118 (2.5)		3.94 (0.64‒24.1)	5/172 (2.9)	2.41 (0.39‒14.9)	2 (5.4)	5.67 (0.70‒45.8)	13/39 (33.3)	7.64 (0.72‒81.53)	<0.001⁎⁎⁎
PD < 28 weeks^a^	0/331	1/118 (0.8)		NA	1/172 (0.6)	NA	0	NA	6/39 (15.4)	NA	<0.001⁎⁎⁎
Spontaneous PD	4/23 (17.4)	3/10 (30.0)		NA	8/30 (26.7)	NA	5/9 (55.6)	NA	2/23 (8.7)	NA	0.063⁎⁎⁎
Iatrogenic PD	19/23 (82.6)	7/10 (70.0)		NA	22/30 (73.3)	NA	4/9 (44.4)	NA	21/23 (91.3)	NA	0.063⁎⁎⁎
Cesarean section^a^	181/332 (54.5)	77/120 (64.2)		1.43 (0.91‒2.24)	113/173 (65.3)	1.37 (0.91‒2.05)	27/37 (73.0)	2.08 (0.89‒4.84)	30/39 (76.9)	2.40 (0.48‒11.96)	<0.001⁎⁎
Intra or postpartum atony	32/357 (9.0)	8/127 (6.3)		NA	17 (9.7)	NA	2/37 (5.4)	NA	7/39 (17.9)	NA	0.27⁎⁎⁎
Delivery HD, days^a^	2.0 (2‒3), *n* = 338	3.0 (2‒3), *n* = 117		1.06 (0.87‒1.29)	3.0 (2‒3), *n* = 157	1.14 (0.97‒1.34)	3.0 (2‒4), *n* = 34	1.22 (0.99‒1.50)	11.5 (5.25‒19.75) *n* = 28	1.66 (1.36‒2.02)	<0.001*
Maternal death	0/357	0/125		NA	0/172	NA	0/37	NA	6/39 (15.4)	NA	<0.001⁎⁎⁎

n, number; M (IQR), Median (interquartile range); PPROM, Preterm Premature Rupture of Membranes; GAD, Gestational Ae at Delivery; PD, Preterm Delivery, HD, Hospital Discharge; NA, Not Applicable; C0, Women with negative serology at delivery and no symptoms during pregnancy (reference group); C1, Women with positive serology at delivery or COVID-19 diagnosis during pregnancy with no symptoms; C2, Women with positive serology at delivery or COVID-19 diagnosis during pregnancy with mild symptoms; C3, Women diagnosed with COVID-19 during pregnancy with hospital admission and moderate symptoms; C4, Women diagnosed with COVID-19 during pregnancy with hospital admission and severe COVID-19.

# The denominator refers to the number of fetuses.

⁎ Kruskal-Wallis non‒parametric test.

⁎⁎ linear-by-linear association.

⁎⁎⁎ Fisher´s exact test.

a Adjusted for confounding factors: gestational diabetes, preterm birth, cesarean section, and days from delivery to discharge.

b Adjusted for confounding factors: fetal distress, oligohydramnios, and fetal death.

**Table 5:** Neonatal outcomes according to maternal COVID-19 infection status.

Outcomes	C0, *n* = 365	C1, *n* = 133		C2, *n* = 181		C3, *n* = 38		C4, *n* = 41		*p*‒value
	Reference	*n* (%), M (IQR)	Adjusted OR (95% CI)	*n* (%), M (IQR)	Adjusted OR (95% CI)	*n* (%), M (IQR)	Adjusted OR (95% CI)	*n* (%), M (IQR)	Adjusted OR (95% CI)	
Birth weight, (g)	3260 (2835‒3615), *n* = 340	3155 (2788‒3555), *n* = 126	NA	3160 (2707‒3447), *n* = 178	NA	3129 (2795‒3574), *n* = 38	NA	2117.5 (1481.25‒3167.5), *n* = 40	NA	<0.001*
Birth weight < 10th percentile#	30/338 (8.9)	12/124 (9.7)	NA	15/177 (8.5)	NA	3/38 (7.9)	NA	0/37	NA	0.37⁎⁎⁎
Birth weight < 3rd percentile#	14/338 (4.1)	2/124 (1.6)	NA	2/177 (1.1)	NA	0/38	NA	0/37	NA	0.22⁎⁎⁎
Apgar score at 5th minute < 7#	5/329 (1.5)	4/118 (3.4)	NA	0/166	NA	1/35 (2.9)	NA	12/34 (35.3)	NA	<0.001⁎⁎⁎
Supplemental oxygen#^,^^a^	36/317 (11.4)	19/119 (16.0)	1.38 (0.64‒2.99)	39/171 (22.8)	1.05 (0.51‒2.15)	6/38 (15,8)	0.54 (0.12‒2.38)	24/36 (66.7)	2.76 (0.93‒8.17)	<0.001⁎⁎
Mechanical ventilation#^,^^a^	2/328 (0.6)	2/123 (1.6)	NA	3/172 (1.7)	NA	3/38 (7.9)	NA	13/36 (36.1)	NA	<0.001⁎⁎⁎
NICU admission#^,^^a^	24/338 (7.1)	13/125 (10.4)	1.24 (0.35‒4.39)	39/177 (22.0)	3.68 (1.43‒9.48)	7/38 (18.4)	5.21 (1.15‒23.67)	23/36 (63.9)	19.36 (5.86‒63.99)	<0.001⁎⁎
Length of hospital stay#^,^^a^	3 (2‒3), *n* = 326	3 (2‒3), *n* = 119	0.99 (0.94‒1.05)	3 (2‒5), *n* = 172	1.01 (0.98‒1.04)	3 (2‒5), *n* = 37	1.01 (0.95‒1.06)	15 (4‒34), *n* = 35	1.02 (0.98‒1.05)	<0.001*
Neonatal death#	0/338	0/125	NA	0/177	NA	0/38	NA	5/40 (12.5)	NA	<0.001⁎⁎⁎

n, number; M (IQR), Median (Interquartile Range); NICU, Neonatal Intensive Care Unit; NA, Not Available due to the small number; C0, Women with negative serology at delivery and no symptoms during pregnancy (reference group); C1, Women with positive serology at delivery or COVID-19 diagnosis during pregnancy with no symptoms; C2, Women with positive serology at delivery or COVID-19 diagnosis during pregnancy with mild symptoms; C3, Women diagnosed with COVID-9 during pregnancy with hospital admission and moderate symptoms; C4, Women diagnosed with COVID-19 during pregnancy with hospital admission and severe COVID-19.

# Denominator refers to the number of neonates.

⁎ Kruskal‒Wallis Non‒Parametric Test.

⁎⁎ Linear-by-linear association.

⁎⁎⁎ Fisher´s Exact Test.

a Adjusted for confounding factors: Apgar score at 5th min <7, supplemental oxygen, mechanical ventilation, NICU admission, and length of hospital stay.

There were no differences in the frequency of preeclampsia and PPROM between the COVID-19 groups (*p* = 0.37 and *p* = 0.06, respectively). Miscarriages were more frequent in the uninfected and asymptomatic infected groups (C0 and C1, respectively) (*p* < 0.001). The rate of fetal distress was significantly higher in the severe COVID-19 group than that in the other groups (31.6% vs. 9.9% in uninfected women; aOR = 4.01; 95% CI 1.84‒8.75). Oligohydramnios was significantly associated with all symptomatic COVID-19 groups (for the mild/C2 group, aOR = 3.77; 95% CI 1.56‒9.07; for the moderate/C3 group, aOR = 6.23; 95% CI 1.93‒20.13; and for the severe/C4 group, aOR = 6.18; 95% CI 1.87‒20.39). A significantly lower mean gestational age at delivery (*p* < 0.001) was observed in the moderate and severe COVID-19 groups. Preterm delivery was more frequent in the moderate and severe COVID-19 groups than that in the other groups at all stratified gestational delivery ages and was associated with moderate (aOR = 3.6; 95% CI 1.45‒9.27) and severe COVID-19 (aOR = 5.51; 95% CI 1.47‒20.61). All maternal deaths (*n* = 6) occurred in the severe COVID-19 group, affecting 15.4% of the women in this group (*p* < 0.001). The postpartum length of hospital stay was significantly longer in the severe COVID-19 group than that in the other groups (11.5 vs. 3 days; aOR = 1.66; 95% CI 1.36‒2.02) (Table 4).

All adverse neonatal outcomes were more frequent in women with severe COVID-19 (*p* < 0.001). While birth weight was nearly 1000 g lower among babies born from mothers with severe COVID-19, there were no differences when stratification was performed for extremely low birth weight, taking into account gestational age at delivery (below 10th percentile, *p* = 0.37; below 3rd percentile, *p* = 0.22). Multivariable adjusted analyses showed that admission to the NICU was the only neonatal complication significantly associated with COVID-19 severity (mild COVID-19, aOR = 3.68; 95% CI 1.43‒9.48) (moderate COVID-10, aOR = 5.21, 95% CI 1.15‒23.67) (severe COVID-19, aOR = 19.36, 95% CI 5.86‒63.99), underscoring the collinearity of many of these outcomes (Table 5).

## Discussion

### Main findings

The findings of the present study showed that COVID-19 influences the rates of adverse obstetric and neonatal outcomes with increasing disease severity. Mild COVID-19 increased the risk of oligohydramnios, and moderate COVID-19 increased the risk of oligohydramnios and preterm birth. These adverse outcomes are also increased in severe COVID-19, which additionally has an increased risk of antepartum fetal distress, longer postpartum hospital stay, and maternal death. For neonates, only the NICU was associated with COVID-19, with progressively increased risk according to severity. Maternal obstetric complications, such as preeclampsia, gestational diabetes, and preterm premature rupture of membranes, were not associated with COVID-19 severity.

### Comparison with results of previous studies

In agreement with previous studies, the authors found that preexisting hypertension and diabetes, as well as higher body mass index, increased the risk of COVID-19 severity.^20^ Conversely, nulliparity decreased the risk of severe COVID-19, which is unrelated to maternal age or maternal comorbidities.

Notably, the frequencies of gestational diabetes were significantly lower in the severe and moderate COVID-19 groups than those in the other groups. This finding may be because women in these two groups were admitted to the hospital due to COVID-19 and delivered prematurely, which could have interfered with the investigation of gestational diabetes. However, a recent systematic review also did not show any significant difference (OR = 1.01; 95% CI 0.86–1.19) in the rates of gestational diabetes among pregnant women affected and unaffected by COVID-19.^15^ On the contrary, Cauldwell et al. (2021) reported an increase of 33.8% in the incidence of gestational diabetes during the COVID-19 pandemic.^21^ The authors postulated that a reduction in the frequency of physical activity in the population may have led to this finding.

The authors observed a higher rate of miscarriages in both uninfected and asymptomatic women. This finding may be because the majority of the present study's patients acquired the disease during the second and third trimesters. Nevertheless, previous studies also did not find an association between miscarriages and COVID-19.^6^^,^^22^ Recently, Sacinti et al. (2021) reported a higher rate of miscarriages in 2020 than in 2019 (RR = 1.25; 1.16–1.35; *p* < 0.0001). However, the rate of positive SARS-CoV-2 test results did not have a significant effect on the miscarriage rate (*p* = 0.810), and the authors believed that this association might be due to the reduction in pregnancy rates.^23^ Studies that include a larger population infected during the first trimester of pregnancy are required to verify these associations.

Hypertensive disorders during pregnancy were not associated with COVID-19 severity in the present study, which is consistent with the findings of a large English cohort study.^24^ However, some studies have reported a higher incidence of preeclampsia in the affected population, both in the presence of the virus^9^^,^^25^ and with worsening maternal conditions.^11^ This hypothesis deserves further investigation since it has been proposed that maternal viral infections may predispose to preeclampsia, both because of inadequate trophoblastic invasion and maternal systematic inflammation.^26^ However, it must be noted that this inflammatory status may lead to a number of features that can mimic a preeclamptic state.^27^

The occurrence of oligohydramnios was more frequent in the present study's population as COVID-19 maternal symptoms progressed, while no difference was found between the uninfected and asymptomatic SARS-CoV-2 seropositive groups. This alteration in amniotic fluid volume may be the consequence of the COVID-19 hypoxemic status that influences the maternal water balance due to dehydration, and in severe cases requiring intensive care, there is a need to control the water balance, which may lead to oligohydramnios. Very few studies have specifically reported the association between amniotic fluid volume and SARS-Cov-2 infection. Soto-Torres et al. (2021), when analyzing ultrasound findings, found no difference in the amniotic fluid index between SARS-CoV-2 positive women and uninfected controls; however, most of the studied population was asymptomatic or presented with mild symptoms.^28^ Similar to the findings of Huntley et al. (2021), the authors observed higher rates of fetal death in severely affected COVID-19 pregnancies, which is related to the severe maternal disease.^2^

A higher incidence of cesarean delivery among women with COVID-19 has been reported,^3^^,^^10^^,^^11^^,^^24^ similar to the authors’ observations. However, Di Toro et al. emphasized that there is no evidence supporting the benefit of this mode of delivery in infected women.^3^ The need for prompt delivery due to the higher frequency of fetal distress, secondary to critical maternal conditions, is a plausible explanation, but probably not the main explanation since even milder cases share this tendency. A systematic review by Debrabandere et al. supports the present finding, suggesting that this may reflect obstetricians’ search for the best possible care, given that the authors currently live in an environment where guidelines and recommendations change constantly.^29^ With advancing knowledge about the COVID-19 effects, the authors expect that rates of the cesarean section will reduce compared with the earlier phases of the pandemic.

The present findings on prematurity are supported by several studies,^6^^,^^9^, ^10^ agreeing that the occurrence of preterm birth rises as maternal conditions worsen. Despite the lack of significant difference between medically indicated delivery according to the severity of COVID-19, the vast majority (91.3%) of preterm deliveries among the group with severe COVID-19 were due to medically indicated delivery. The authors believe that this lack of significance could be due to the small number of cases in the moderate COVID-19 group. The rates of preterm deliveries before 37, 34, and 32 weeks of gestation were significantly higher, not only in severely affected women but also in those with moderate symptoms. Although some studies^15^^,^^30^ have reported a decreasing rate of prematurity in high-income countries during this period, possible biases cannot be excluded since changes in lifestyle, such as better nutrition, reduced work-related stress situations, and fewer lockdown measures, have probably resulted in the reduction in emergency care consultations for reasons that could culminate in medically indicated preterm births.

Regarding neonatal outcomes, admission to the NICU was associated with disease severity in the mother. Indeed, lower Apgar scores at five minutes, need for supplemental oxygen, and mechanical ventilation was also correlated with NICU admission and were not significant as isolated variables. In addition to prematurity, another factor that may influence the high risk of NICU admission in severe COVID-19 is the use of drugs for maternal muscular relaxation and sedation in the ICU, which has an impact on the first minutes of life. It is important to distinguish these adverse neonatal outcomes as a consequence of prematurity rather than the direct effect of the virus on neonates.^10^

### Strengths and limitations

The main strengths of this study include its relatively large sample size for a single-center study conducted under harmonized study and management protocols. The addition of severity category groups and a control group allowed for a specific determination of the effect of increasingly severe COVID-19 on the study outcomes, which very few studies have examined. This information will allow more nuanced recommendations to obstetricians and mothers regarding prevention through vaccination and management of severe COVID-19 as a key factor influencing severe adverse pregnancy outcomes.

A limitation of the study could be that it covered a long period of time, and the authors did not investigate the SARS-CoV-2 genotype, as new strains were recognized during this period.

### Implications for research and clinical practice

One of the most frequent concerns of medical professionals and pregnant women is whether the acquisition of the COVID-19 virus alone causes gestational complications or if the impact is directly related to the maternal well-being damage. The present study is a step towards better understanding this concern.

Professionals dealing with pregnant women must be aware of severe and symptomatic diseases. Asymptomatic infection does not add additional risk compared with the absence of infection; in this regard, vaccination during pregnancy should be recommended to prevent serious disease.

## Conclusion

Adverse and neonatal outcomes are associated with maternal symptomatic COVID-19 status, and the risk increases with disease severity.

## Funding

This work was supported by CAPES (88881.504727/2020-01) and the European Union's Horizon 2020 Research and Innovation Programme under ZIKAlliance Grant Agreement nº 734548. The funders had no role in the study design, data collection and analysis, decision to publish, or manuscript preparation.

## Declaration of Competing Interest

The authors declare no conflicts of interest.
